# FtsZ contributes to cytoadhesion and interaction with host extracellular matrix components and plasminogen in *Mycoplasma bovis*

**DOI:** 10.1186/s13567-025-01673-y

**Published:** 2025-12-12

**Authors:** Shanyu Jin, Shengli Chen, Huafang Hao, Shimei Lan, Xiangrui Jin, Zhangcheng Li, Xinyi Wang, Yifan Zhang, Xinmin Yan, Yuefeng Chu

**Affiliations:** 1 State Key Laboratory for Animal Disease Control and Prevention, College of Veterinary Medicine, Lanzhou University, Lanzhou Veterinary Research Institute, Chinese Academy of Agricultural Sciences, Lanzhou, 730000 China; 2 Gansu Province Research Center for Basic Disciplines of Pathogen Biology, Lanzhou, 730046 China; 3 Key Laboratory of Veterinary Etiological Biology, Key Laboratory of Ruminant Disease Prevention and Control (West), Ministry of Agricultural and Rural Affairs, Lanzhou, 730046 China

**Keywords:** *Mycoplasma*, *Mycoplasma bovis*, FtsZ, extracellular matrix, plasminogen

## Abstract

*Mycoplasma bovis* causes serious diseases in cattle and is one of the most economically important pathogens threatening the global cattle industry. It is still a great challenge to prevent and control *M. bovis* infections owing to the increase in antibiotics resistance and unsatisfactorily effective commercial vaccines. FtsZ is commonly considered as a division protein in most bacteria. Owing to the limited genetic manipulation tools of *M. bovis*, the role of FtsZ in the pathogenicity of *M. bovis *remains unknown. In this study, FtsZ protein was found to be mainly distributed on the cell membrane, was highly conserved, and had good immunogenicity. FtsZ could bind to embryonic bovine lung cells (EBL) membrane extract, and this binding was inhibited by anti-FtsZ serum. In addition, FtsZ interacted with host extracellular matrix components fibronectin, laminin, vitronectin, and collagen IV in a dose-dependent manner, and promoted tissue diffusion by activating plasminogen through tissue plasminogen activator (tPA). Disruption of the *ftsZ* gene markedly reduced the adhesion of *M. bovis* to EBL cells while the complemented strain *M. bovis* Δ*ftsZ*: *ftsZ* partly restored the adhesion ability. This is a first description that FtsZ acts as a novel adhesion protein in *Mycoplasma*, which interacts with host extracellular matrix components and plasminogen, and plays a vital role in adhesion and dissemination. This study expands our understanding of FtsZ significant role in the pathogenicity of *M. bovis* and provides a new potential vaccine and drug target against *M. bovis* infections.

**Supplementary Information:**

The online version contains supplementary material available at 10.1186/s13567-025-01673-y.

## Introduction

*Mycoplasma* are the smallest prokaryotic microorganisms that are capable of independent replication. They have no cell wall, diverse morphology, widely exist in nature, and many of them are pathogenic for humans and livestock [1]. *Mycoplasma bovis* (*M. bovis*), first isolated from a cow with mastitis in the USA in 1961 [2], is one of the most important pathogens in cattle. It can cause pneumonia, arthritis, mastitis, otitis media, and keratoconjunctivitis in cattle [3]. *M. bovis* infections are distributed all over the world, and both beef and dairy cattle are susceptible to it. *M. bovis* has a very small genome, low G + C content (about 29%) [4], and simple energy metabolism [5]. It causes serious disease and considerable economic losses to the animal husbandry industry. The prevention and control of *M. bovis* infection is a great challenge owing to the increase in antibiotics resistance [6] and the unsatisfactory efficacy of commercial vaccines.

Adhesion is a prerequisite for pathogenic bacteria to colonize and infect host cells [7]. Owing to the lack of a cell wall and no terminal organelles with adhesion function such as *Mycoplasma pneumoniae* (*M. pneumoniae*) [8] and *Mycoplasma genitalium* (*M. genitalium*) [9], the cell membrane and membrane-associated proteins of *M. bovis* are the key factors for adhesion and virulence [10]. Membrane-associated proteins and glycolipids are regarded as the main antigens of *M. bovis*, which can stimulate the host to produce humoral and cellular immune responses and induce inflammatory responses [11]. The target proteins of *M. bovis* adhesion to host cells are mainly extracellular matrix components (ECM), including collagen, elastin, fibronectin, platelet-derived growth factor, and laminin [12], etc. However, most pathogens produce low levels of protease and have a limited ability to degrade ECM components, while plasminase can efficiently degrade ECM proteins, which constitute a key tissue barrier. Many bacteria have been found to promote plasminogen activation into plasmin [13]. Previous studies have reported that variable surface protein (Vsp), α-enolase, Mbov0503, Fructose-1,6-bisphosphate aldolase, mbfN, MilA, and LppA are adhesive-related proteins of *M. bovis* [12, 14–19]. There is still an urgent need to identify and investigate other potential candidate targets for *M. bovis* infection*.*

In our previous high-throughput screening, we found that the ability of *M. bovis* Δ*ftsZ* mutant to adhere to EBL cells decreased significantly, indicating its potential adhesion role in the *M. bovis* [20]. FtsZ is highly conserved in bacteria [21] and it is commonly considered as a division protein in most bacteria, such as *Escherichia coli*, *Staphylococcus aureus*, *Bacillus subtilis* [22]. At present, the function of FtsZ in *M. bovis* remains unknown.

In this research, the role of FtsZ in the adhesion of *M. bovis* to host cells at the protein and bacterial levels and its immunogenicity are investigated. These collectively will unveil FtsZ role in the adhesion and infection of *M. bovis* and its potential new therapeutic and vaccine targets.

## Materials and methods

### Bacterial strain and cell culture

*M. bovis* reference strain PG45 and six Chinese clinically isolated strains were propagated in a pleuropneumonia-like organism medium supplemented with 10% horse serum (Gibco) (Additional file 1) [23]. *M. bovis* Δ*ftsZ* mutant required an additional kanamycin (100 μg/mL), and the complemented strain was generated by transforming Δ*ftsZ* mutant with pOH-*ftsZ* plasmid, with 10 μg/mL puromycin in the medium to maintain selection pressure. Embryonic bovine lung (EBL) cells were cultured in Dulbecco’s modified eagle medium (DMEM) (Gibco, USA) as previously described [12]. *Escherichia coli* DH5α and BL21 (DE3) strains cultured in Luria–Bertani (LB) medium, were used for gene cloning and protein expression, respectively.

### DNA constructs and transformation

The complete sequence of *M. bovis ftsZ* gene (MBOVPG45_0460) was optimized for *E. coli*-preferred codons and synthesized by Sangon Biotech to generate plasmid pET-30a-*ftsZ*. The prokaryotic expression plasmid pET-30a-*ftsZ* was introduced into *E. coli* strain BL21(DE3) through heat shock. Plasmid pOH/P [24], which encoded a puromycin N-acetyltransferase [25], was used as a skeleton for constructing complementation plasmid pOH-*ftsZ* (Additional file 1). The resulting complementation plasmid was introduced into the Δ*ftsZ* mutant by PEG 8000, generating the complemented Δ*ftsZ*:*ftsZ* strain. To confirm the successful construction of the *M. bovis* Δ*ftsZ:ftsZ*, genomic DNA was extracted from *M. bovis* PG45, *M. bovis* Δ*ftsZ* and *M. bovis* Δ*ftsZ:ftsZ* using a bacterial genomic DNA Extraction Kit (TIANGEN, China) and was used as a template for PCR amplification and primers pOH-*ftsZ*-F/R (Additional file 1) was used. The PCR program was as follows: 94 °C for 3 min; followed by 35 cycles of 94 °C for 30 s, 50 °C for 30 s, and 72 °C for 60 s, final extension by 72 °C for 5 min. The amplified products were analyzed by 1.0% agarose gel electrophoresis.

### Preparation of recombinant protein and polyclonal antibodies

Recombinant protein rFtsZ expression was induced by using isopropyl β-d-thiogalactoside and purification by Ni–NTA resin (GenScript Biotech Corporation, China) as previously described [12]. Polyclonal antibodies against rFtsZ was produced by immunizing 6-week-old specific pathogen-free (SPF) BALB/c mice. Each mouse was immunized three times with 20 μg of rFtsZ mixed with QuickAntibody-Mouse 5W adjuvant (KX0210041, Biodragon, Beijing, China) (1:1) by intramuscular injection of the leg  on day 21 and day 35 after first immunization. Antisera were collected at 2 weeks after the third immunization, and the antibody titer was assessed using enzyme-linked immunosorbent assay (ELISA).

### Subcellular localization of FtsZ in *M. bovis*

The subcellular localization of FtsZ was explored using colony blot and western blot assays as as previously described [20]. For colony blot assay, polyvinylidene fluoride (PVDF) membranes were gently placed on *M. bovis* colonies on the surface of agar plates. After transferring *M. bovis* colonies to the membrane, anti-FtsZ mouse serum or preimmune serum (1:1000) was used as the primary antibody, and horseradish peroxidase (HRP)-labeled goat anti-mouse IgG (H + L) was used as the secondary antibody. For western blot assay, cell membrane and cytoplasmic proteins of *M. bovis* PG45 were extracted using a cell membrane extraction kit (89842, Thermo). The extracts were separated using sodium dodecyl sulfate–polyacrylamide gel (SDS-PAGE) and western blot was conducted. Anti-FtsZ mouse serum (1:1000) was used as the primary antibody, and HRP-labeled goat anti-mouse IgG (H + L) (A0216, Beyotime Biotechnology, Shanghai, China) (1:1000) was used as the secondary antibody.

### Mycoplasma adhesion assay

The EBL cells were cultured into 12-well plates and incubated overnight at 37 °C in a 5% CO_2_ atmosphere. Subsequently, the EBL cells were infected with *M. bovis* PG45, *M. bovis* Δ*ftsZ*, and *M. bovis* Δ*ftsZ:ftsZ* at a multiplicity of infection (MOI) of 1000 for 1.5 h at 37 °C. The adhesion rate was calculated [12]. For adhesion inhibition assay, heat-inactivated anti-FtsZ serum was preincubated with *M. bovis* cells at 37 °C for 1 h, while preimmune serum was utilized as a negative control.

### Fluorescence-based adhesion assay

rFtsZ protein were added to EBL cells in a 24-well plate at 37 ℃ for 40 min. EBL cells were then fixed with 4% paraformaldehyde and permeabilized with 0.1% Triton X-100. Fluorescence-based adhesion test was performed as previously described [20]. The proteins was labeled using goat anti-mouse IgG (H + L) Alexa Fluor^TM^ 488 (A-11029, Thermo), the cell membrane of EBL cells was labeled using 1,1’-dioctadecyl-3,3,3’,3’-tetramethylindocarbocyanine perchlorate (DiI) (C1991S, Beyotime Biotechnology, Shanghai, China), and the cell nuclei was labeled using 4’,6-diamidino-2-phenylindole (DAPI) (MBD0015, Sigma). Images were captured using a Carl Zeiss Laser Scanning Microscope LSM980. For *Mycoplasma* adhesion assays, *M. bovis* PG45, *M. bovis* Δ*ftsZ* and *M. bovis* Δ*ftsZ*:*ftsZ* were labeled using CFDA-SE (C0051, Beyotime Biotechnology, Shanghai, China) as previously described [19].

### Western blot

Protein samples were separated on 10% SDS-PAGE and transferred to PVDF membrane (Amersham). The membrane was blocked with 5% BSA at room temperature for 2 h and then incubated with the experimental infected serum (28 days of postinfection for *M. bovis* 13690 strain) or vaccinated calves serum or anti-FtsZ mouse serum as primary antibody at 4 °C overnight or room temperature for 2 h. After washing with TBST (0.05% Tween-20 in Tris-buffered saline, 10 mM Tris, 150 mM NaCl, pH 7.4), The membrane was incubated at room temperature with 1:1000 HRP-labeled goat anti-mouse IgG (H + L) (Beyotime Biotechnology, Shanghai, China) or 1:5000 HRP-labeled goat anti-cow IgG (H + L) (Abcam) for 1 h. The image detection was performed using ECL (Thermo Fisher Scientific) with a ChemiDoc XR + image system (Bio-Rad).

### Dot blot

Serial two-fold dilutions of rFtsZ protein (2.0~0.125 μg) were applied onto nitrocellulose (NC) membranes (Solarbio), 6 × His peptides was applied as negative control. After drying, NC membranes were blocked with QuickBlock™ Western (P0252, Beyotime Biotechnology, Shanghai, China) for 1 h at room temperature. The membranes were then incubated overnight at 4 °C with 10 μg of fibronectin, laminin, vitronectin, collagen IV, tissue plasminogen activator (tPA), or plasminogen, respectively. After washing, primary antibodies such as anti-fibronectin (rabbit polyclonal antibody, Bioss antibodies), anti-laminin (rabbit monoclonal antibody, abcam), anti-vitronectin (rabbit monoclonal antibody, abcam), anti-collagen IV (rabbit monoclonal antibody, abcam), anti-tPA (rabbit monoclonal antibody, abcam), or anti-plasminogen (rabbit polyclonal antibody, Solarbio) were added and incubated for 1 h at room temperature. Goat anti-mouse HRP-IgG or goat anti-rabbit HRP-IgG was then applied as secondary antibody and incubated together at room temperature for 1 h, followed by enhanced chemiluminescence (ECL) detection. Membranes were visualized using a ChemiDoc XR + image system (Bio-Rad) [12].

### ELISA

EBL cell membrane protein or ECM components (fibronectin, laminin, vitronectin, and collagen IV) were coated onto 96-well plate at 4 °C overnight, which were subsequently blocked with 5% skim milk, and serial two-fold dilutions of rFtsZ solution were added to the wells; mouse anti-FtsZ serum was applied as the primary antibody and goat anti-mouse HRP-IgG as the secondary antibody. After addition of a 3,3′, 5,5′-tetramethylbenzidine (TMB) substrate, absorbance was measured using the iMark microplate reader (Bio-Rad) [12].

### Plasminogen activation assay

The plasminogen activation test was performed as previously described [12]. rFtsZ protein (1 μg) was incubated with plasminogen (1 μg) for 1 h at 37 °C and then added to 96-well plates. Subsequently, tPA (50 ng) or the lysine analog aminoacetic acid ε-ACA (80 mM) was added, and the mixture was incubated at 37 °C for 15 min. After adding 0.5 mM of the specific substrate D-Val-Leu-Lys *p*-nitroaniline dihydrochloride (V7127, Sigma), the absorbance changes at 405 nm were monitored at different time points using the iMark microplate reader.

### Phylogenetic tree and bioinformatics analyses of FtsZ

The NCBI database was utilized to search for the FtsZ protein sequence of *M. bovis* (Additional file 2) and other mycoplasma species. The amino acid sequence identity were analyzed using the Basic Local Alignment Search Tool (Blastp). Sequence alignment were performed through ClustalW and visualization with Esprit 3.0 [26], A phylogenetic tree was constructed using the MEGA 12.0 software [27], and visualization was performed using iTOL tool [28].

### Statistical analysis

All statistical analyses were performed using GraphPad Prism version 8.0. Multiple comparisons were performed using an unpaired *t*-test or one/two-way analysis of variance (ANOVA) for the analysis of studies where appropriate. All values were expressed as the means ± standard deviations as indicated. Significant differences were indicated as follows: *p < 0.05, **p < 0.01, ***p < 0.001. ****p < 0.0001. ns indicated no statistical significance (*p* > 0.5).

## Results

### Sequence analyses of FtsZ

**Figure 1:**
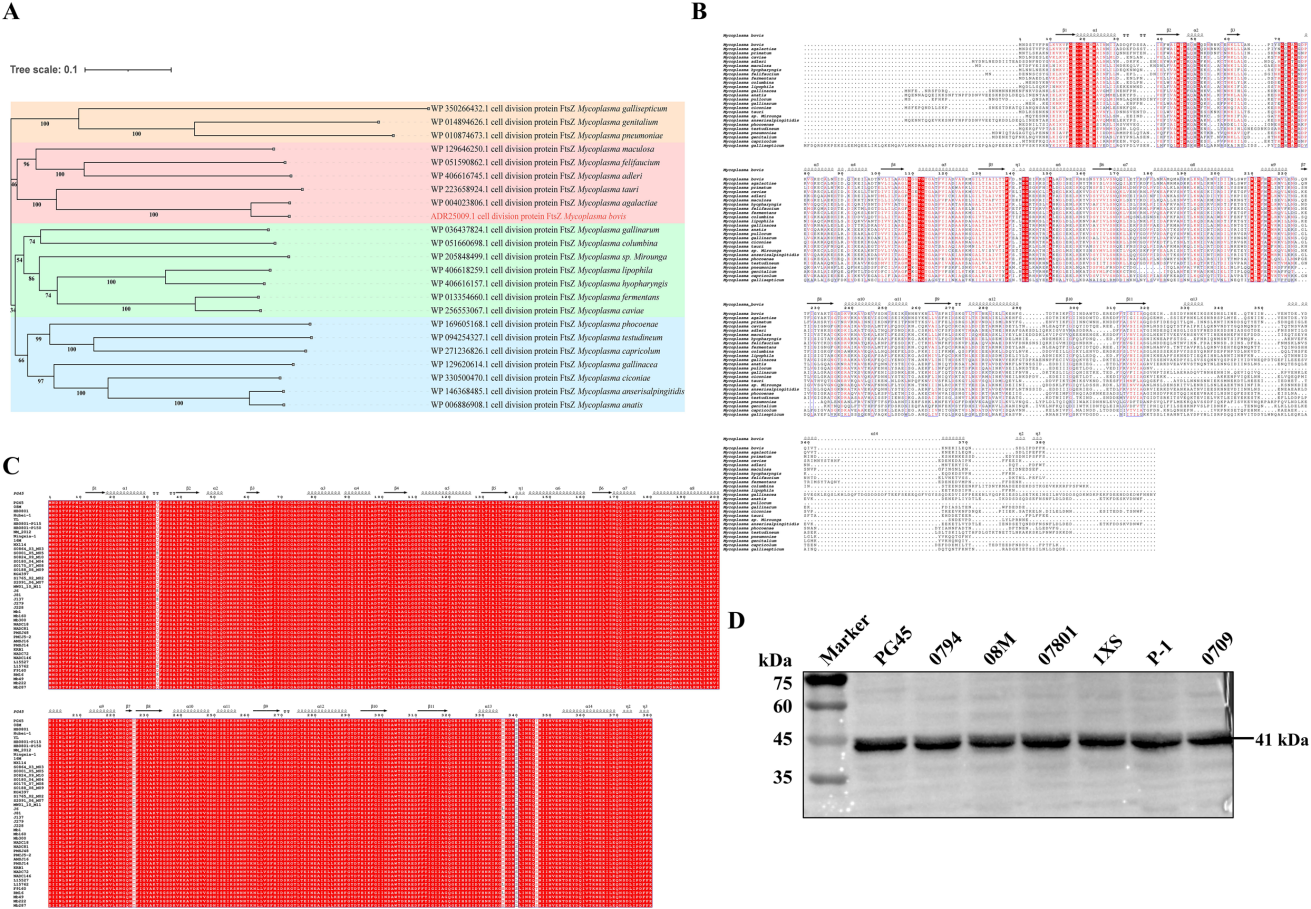
**Bioinformatics analyses of FtsZ amino acid sequence.** **A** Phylogenetic tree analysis of the distribution and genetic distance of FtsZ protein in *Mycoplasma*. The FtsZ of *Mycoplasma* could be classified into different groups according to its topological structure. Different background colors represent different groups. **B** Multiple alignment of the amino acid sequences of the FtsZ of *Mycoplasma*. Identical residues are depicted in white letters with red background, while similar residues are in red letters with white background. The predicted secondary structures of *M. bovis* PG45 FtsZ are displayed above the corresponding aligned regions, *α* represents *α*-helix, *β* represents *β*-sheet, *η* represents coil, and *T* represents turn. **C** Multiple conserved sites of FtsZ in *M. bovis*. **D** Expression situation of FtsZ in different *M. bovis* strains. Whole-cell proteins of PG45 and six other strains of *M. bovis* were separated by sodium dodecyl sulfate–polyacrylamide gel (SDS-PAGE), transferred onto polyvinylidene fluoride (PVDF) membrane, incubated in anti-FtsZ serum (1:1000), and then probed with HRP-labeled goat anti-mouse IgG (1:1000).

*M. bovis* FtsZ had amino acid sequence identity ranging from 30.52% to 91.05% with other *Mycoplasma* species. A phylogenetic tree based on the amino acid sequence of FtsZ in *Mycoplasma* was established. According to the layout of phylogenetic tree, five groups were divided, among which *M. bovis* and *M. agalactiae* were closely clustered together, while FtsZ in *M. pneumoniae*, *M. genitalium*, and *M. gallisepticum* were closely clustered (Figure 1A). Further multiple sequences alignment analysis revealed multiple highly conserved sites that may play crucial role in the biological function of FtsZ (Figure 1B). Subsequently, multi-sequence alignments among *M. bovis* strains were conducted, and FtsZ exhibited highly conserved among strains isolated from different countries, host species, and isolation sites (Figure 1C) (Additional file 2), with sequence identity reaching over 99%. In addition, we conducted western blot analyses on the protein extracts of six clinical *M. bovis* isolates and the reference strain PG45, FtsZ protein expressed in all examined strains (Figure 1D). This indicates that FtsZ is highly conserved in different isolates of *M. bovis* and can be accurately expressed. In addition, no FtsZ-related homologous were found in the bovine genome (NCBI blastp). The above analysis indicates that FtsZ may be a potential vaccine candidate used to develop broad-spectrum vaccines against *M. bovis*.

### FtsZ is an immunogenic protein

**Fig. 2:**
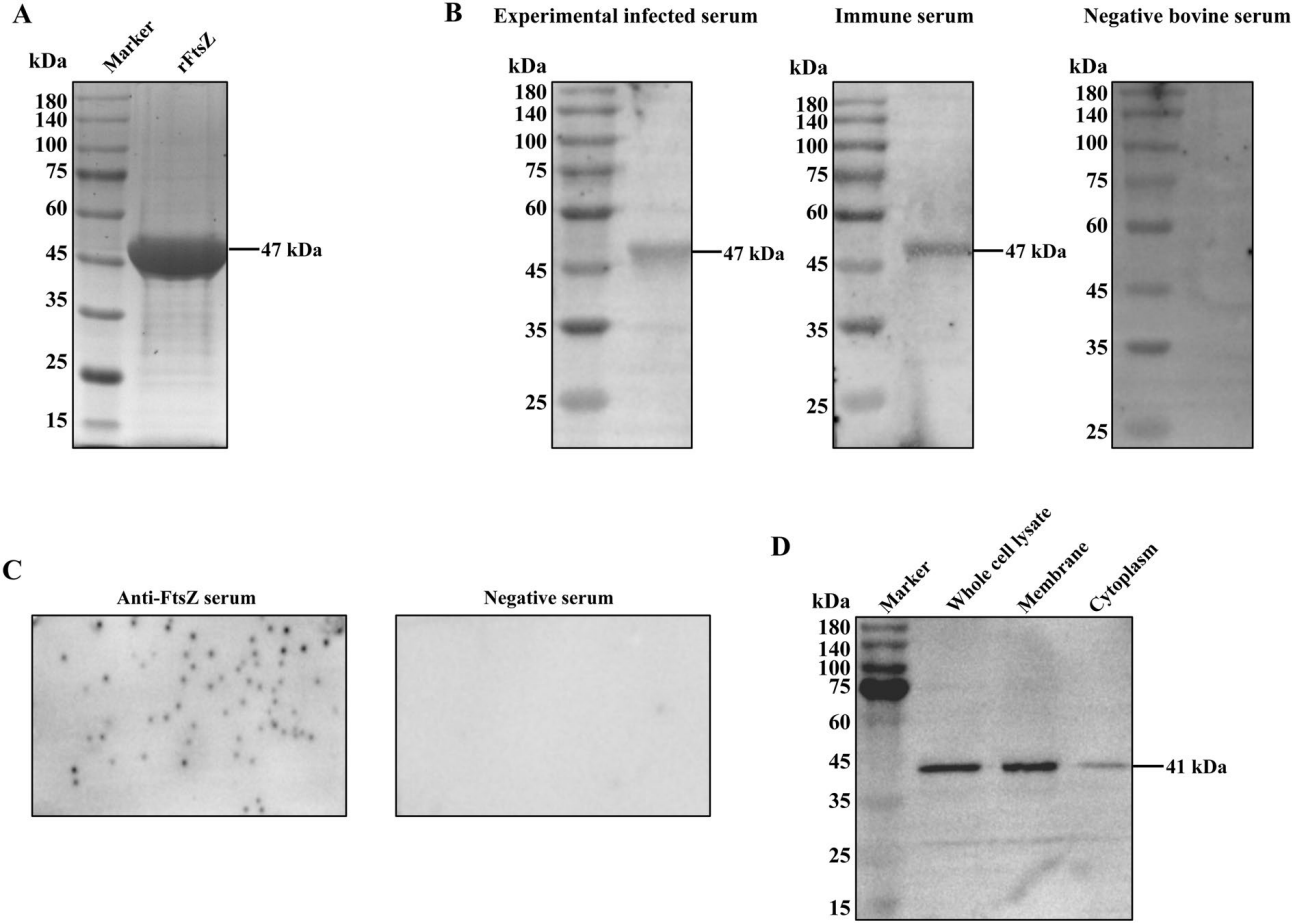
**Immunogenicity and subcellular location of FtsZ in** ***M. bovis***. **A** Purification of recombinant protein rFtsZ. The proteins purified by Ni–NTA column were separated using 10% SDS-PAGE. **B** Immunogenicity of FtsZ protein in *M. bovis*. The rFtsZ were transferred to the PVDF membrane, the membrane was incubated with healthy calve serum (1:50), experimental infection of calve serum with *M. bovis* (1:50) and immune calve serum (1:50), respectively, and then probed with HRP-labeled goat anti-cow IgG (1:5000). **C** Colony blot assay of FtsZ in *M. bovis*, after transferring *M. bovis* colonies to a PVDF membrane, anti-FtsZ mouse serum, or pre-immune serum (1:1000) was used as the primary antibody, and HRP-labeled goat anti-mouse IgG (H + L) was used as the secondary antibody. **D** Surface localization of FtsZ in *M. bovis*. The whole cell protein, membrane protein, and cytoplasmic protein of *M. bovis* PG45 were extracted and transferred to PVDF membrane, incubated with mouse anti-FtsZ serum (1:1000), and then probed with HRP-labeled goat anti-mouse IgG (1:1000).

rFtsZ was mainly expressed as a soluble protein of about 47 kDa in recombinant *E. coli* BL21(DE3), detected by SDS-PAGE analysis (Figure 2A).Western blot analysis showed that *M. bovis* FtsZ protein could react experimentally with *M. bovis* infected and immunized calves serum, but not with *M. bovis* negative bovine serum (Figure 2B). Therefore, *M. bovis* FtsZ can stimulate the production of specific antibodies in cattle.

### FtsZ is mainly localized in the cell membrane in *M. bovis*

The distribution of FtsZ protein in *M. bovis* cells was analyzed using colony blot assay. The anti-FtsZ serum reacted with the *M. bovis* colonies, while no hybridization spots were obtained after incubating with the negative serum (Figure 2C). It indicates that FtsZ protein is expressed on the surface of *M. bovis*. To further confirm the localization of FtsZ, whole cell lysate, membrane proteins, and cytoplasmic proteins of *M. bovis* cells were detected with anti-FtsZ serum. The result confirmed that FtsZ is mainly localized in the cell membrane, with small proportions in the cytoplasm (Figure 2D).

### FtsZ adheres to host EBL cells

**Figure 3:**
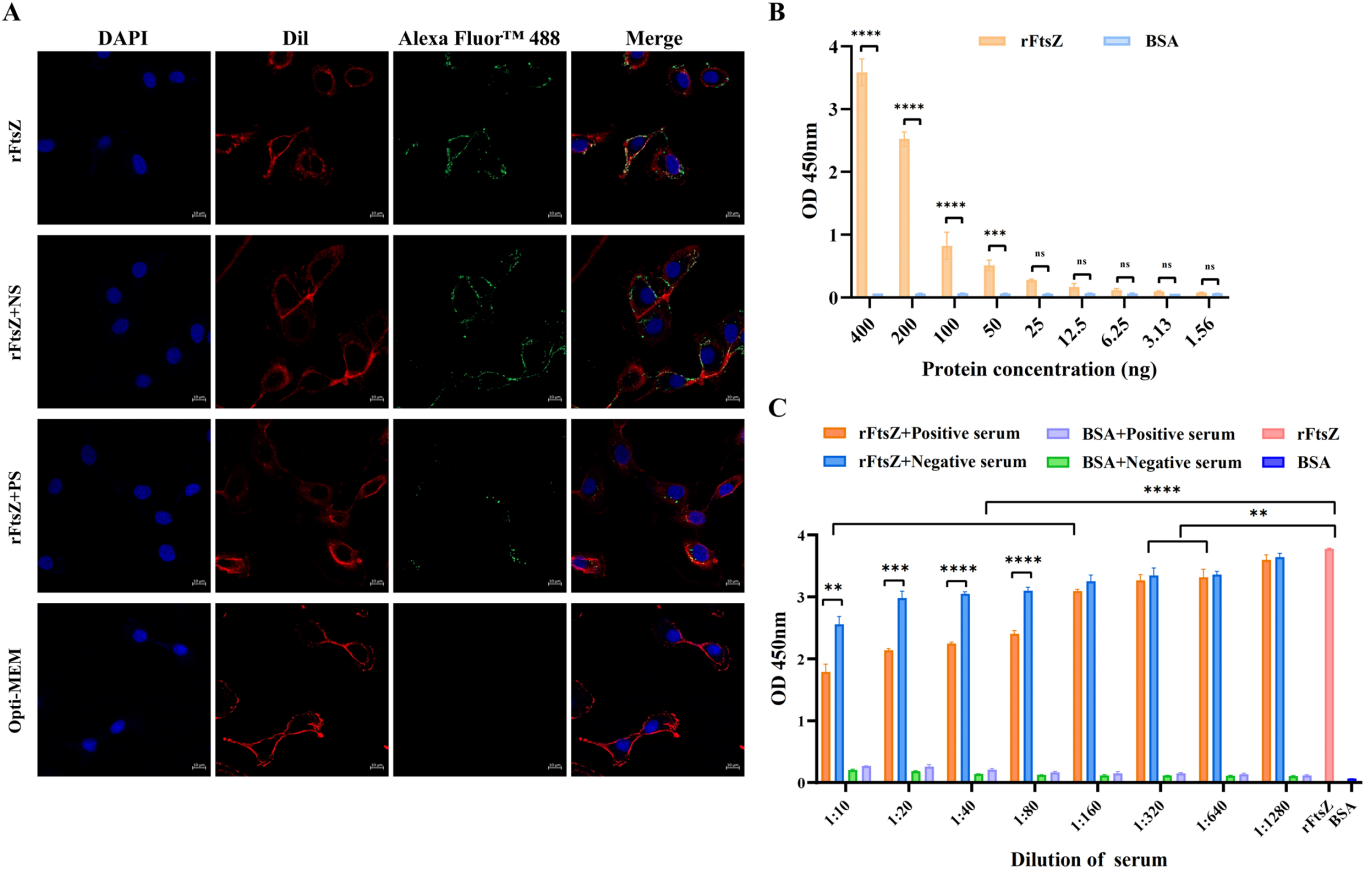
**Interaction between FtsZ of** ***M. bovis*** **and embryonic bovine lung (EBL) cells**. **A** The adhesion of FtsZ to EBL cells was observed by laser confocal microscopy. A total of 150 μg rFtsZ was preincubated with positive (rFtsZ + PS) or negative serum (rFtsZ + NS) for 1 h, then tested with anti-FtsZ serum (1:100) and goat anti-mouse IgG (H + L) Alexa Fluor™ 488 (green, 1:200). The cell membrane (red) and nucleus (blue) were labeled with DiI and DAPI, respectively. Opti-MEM group was used as a control. (scale bar = 10 µm). **B** FtsZ binds to EBL cell membrane extracts in a dose-dependent manner (ranged from 1.56 ng to 400 ng). The 400 ng EBL membrane extracts were incubated with serial dilutions of rFtsZ or BSA in 96-well plate. Mouse anti-FtsZ serum was used as the primary antibody and HRP-labeled goat anti-mouse IgG for the secondary antibody. **C** Binding inhibition assay. rFtsZ or BSA was preincubated with serial dilutions of anti-FtsZ serum and negative serum (1:10 ~ 1:1280) before incubation with EBL membrane extracts. Values are expressed as mean ± standard error of three replicates (**p < 0.01, ***p < 0.001, ****p < 0.0001, ^ns^*p* > 0.5).

The function of FtsZ in adhesion of *M. bovis* to host EBL cells was evaluated by laser confocal microscopy, which revealed that rFtsZ could significantly adhere to EBL cells. When the recombinant protein was preincubated with anti-FtsZ serum, the adhesion of rFtsZ to EBL cells was greatly reduced, while mouse negative serum did not show a significant inhibitory effect (Figure 3A). It was showed that rFtsZ could bind to cell membrane proteins in a dose-dependent manner in the range of 25–400 ng, compared with BSA control in the ELISA assay (Figure 3B). This binding was significantly inhibited by anti-FtsZ antibodies at a dilution of 1:10 to 1:640 while a certain inhibitory effect for negative serum (Figure 3C). Hence, *M. bovis* FtsZ binds directly to the host cell surface.

### FtsZ interacts with host ECM components

**Figure 4:**
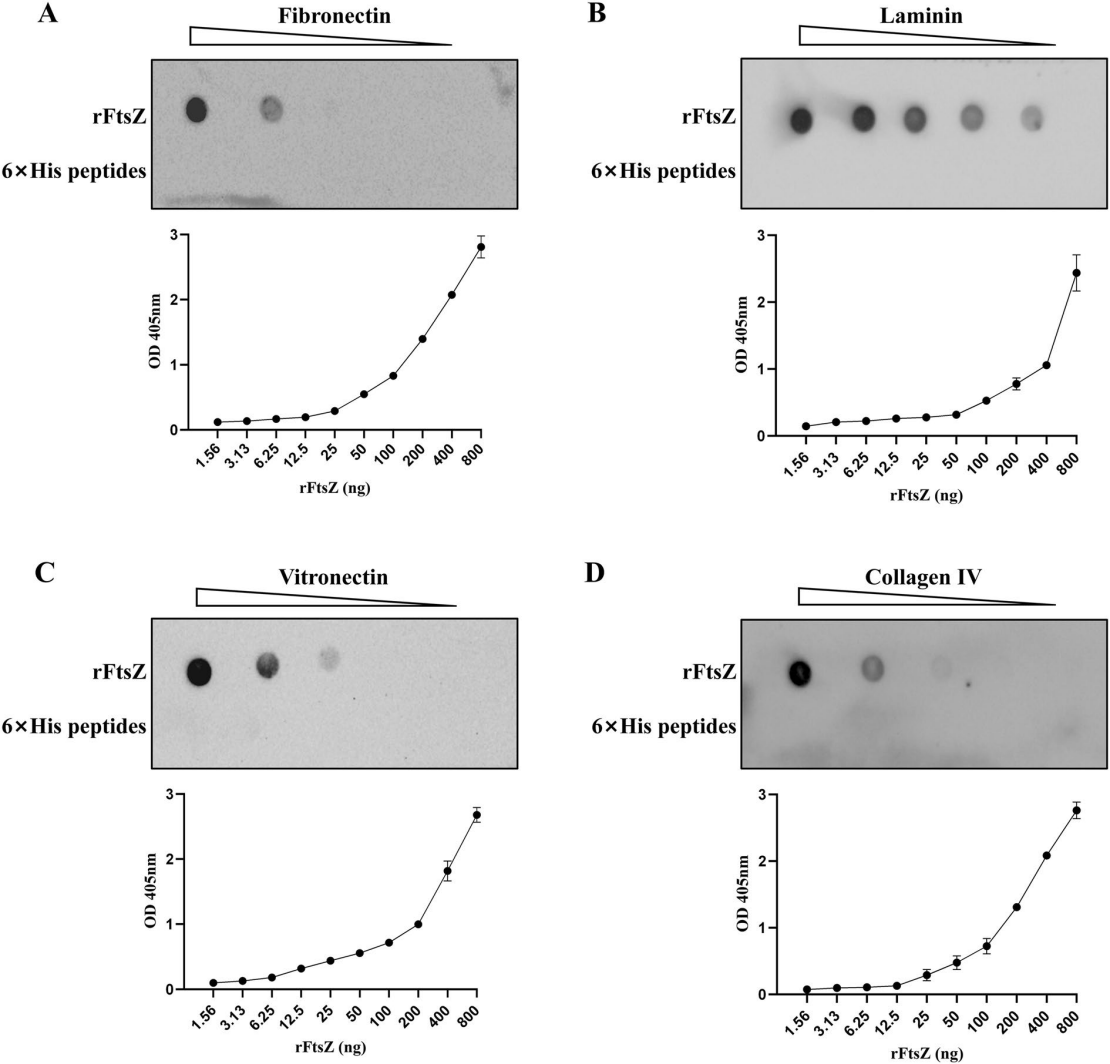
**FtsZ of** ***M. bovis*** **binds to the host extracellular matrix (ECM) components in a dose-dependent manner**. **A** The detection of FtsZ binding to fibronectin. For dot blot test, serial twofold dilutions of rFtsZ (0.125–2 μg) were spotted onto a nitrocellulose membrane and then incubated with 10 μg fibronectin. The 6 × His peptides as the negative control. For ELISA test, different concentrations of rFtsZ (1.56–800 ng) were added to wells coated with fibronectin (200 ng). The optical density (OD) value was measured at 405 nm. **B** The detection of rFtsZ binding to laminin. **C** The detection of rFtsZ binding to vitronectin. **D** The detection of rFtsZ binding to collagen IV.

To further investigate the adhesion properties of FtsZ to host cells and to confirm whether FtsZ could interact with host ECM components, dot blot and ELISA tests were performed. It was found that rFtsZ could bind to fibronectin, laminin, vitronectin, and collagen IV in a dose-dependent manner (Figure 4).

### FtsZ promotes the conversion of plasminogen to plasmin through tPA

**Figure 5:**
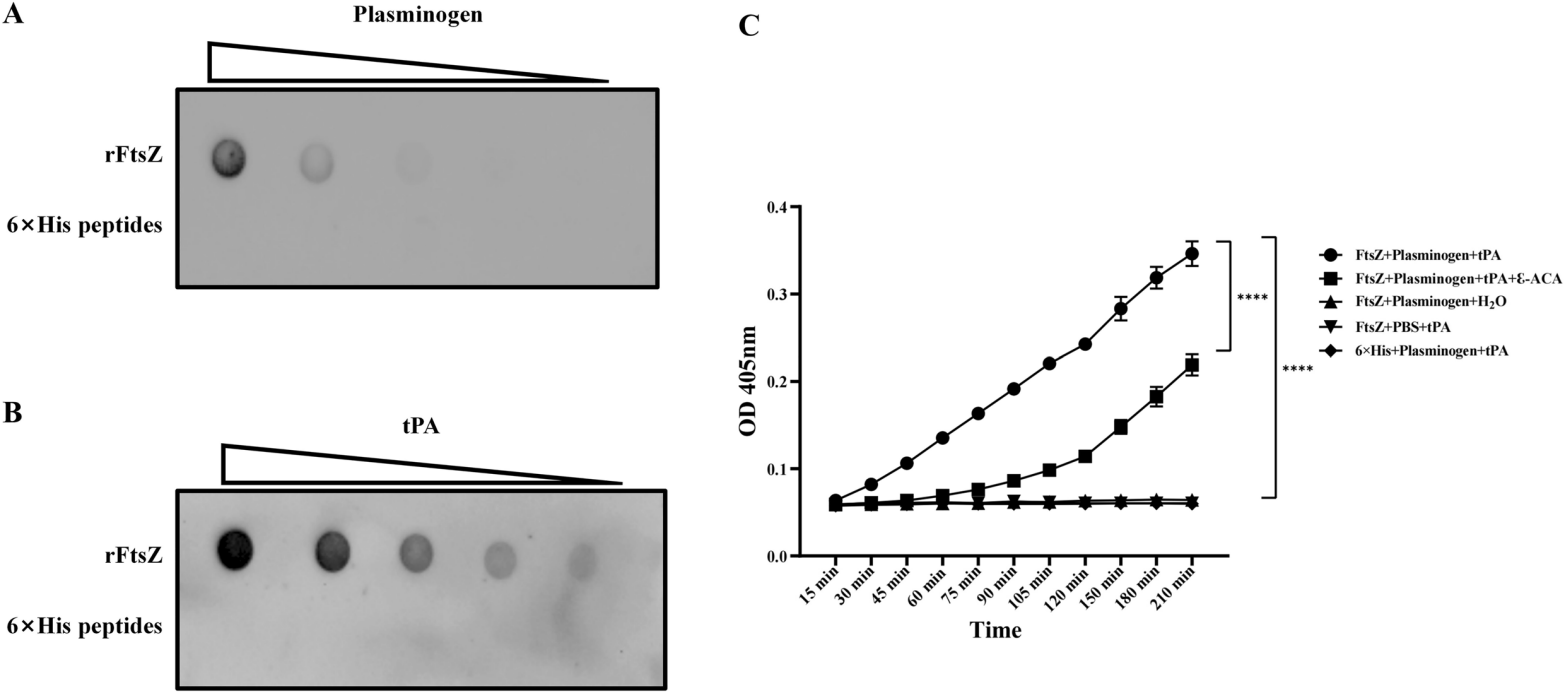
**FtsZ promotes the activation of plasminogen by tPA.** **A, B** Dot blot analysis of the binding ability of rFtsZ to plasminogen and tPA. The 6 × His peptides as the negative control. **C** Kinetic curves of FtsZ promoted plasminogen activation by tPA. Plasminogen and rFtsZ were incubated on a microtiter plate for 1 h, and then tPA and the specific substrat d-Val-Leu-Lys *p*-nitroaniline dihydrochloride were added. Plasmin activity was detected by OD value at 405 nm. Wells without plasminogen or tPA were used as negative controls.

As shown in Figures 5A, B, rFtsZ could bind plasminogen and tPA. Plasminogen activation was then detected using the plasmin-specific chromogenic substrate D-Val-Leu-Lys *p*-nitroaniline dihydrochloride. The activity of wells containing rFtsZ protein, plasminogen, and tPA was detected with an OD 405 nm, which was significantly higher than that of 6 × His peptides-coated wells (Figure 5C). The results indicated that rFtsZ improved the efficiency of the conversion of plasminogen to plasmin. Incubation with the lysine analogue ε-ACA resulted in a significant decrease of OD values in wells containing rFtsZ, tPA, and plasminogen (Figure 5C), indicating that ε-ACA affected the ability of rFtsZ to activate plasminogen. However, the wells containing only plasminogen and rFtsZ or rFtsZ and tPA found no increased OD value, suggesting that rFtsZ only strengthened the plasminogen activation through tPA, and did not replace the tPA or plasminogen function.

### Disruption of *ftsZ* gene significantly reduces the adhesion capacity of *M. bovis* to host cells in vitro

**Figure 6:**
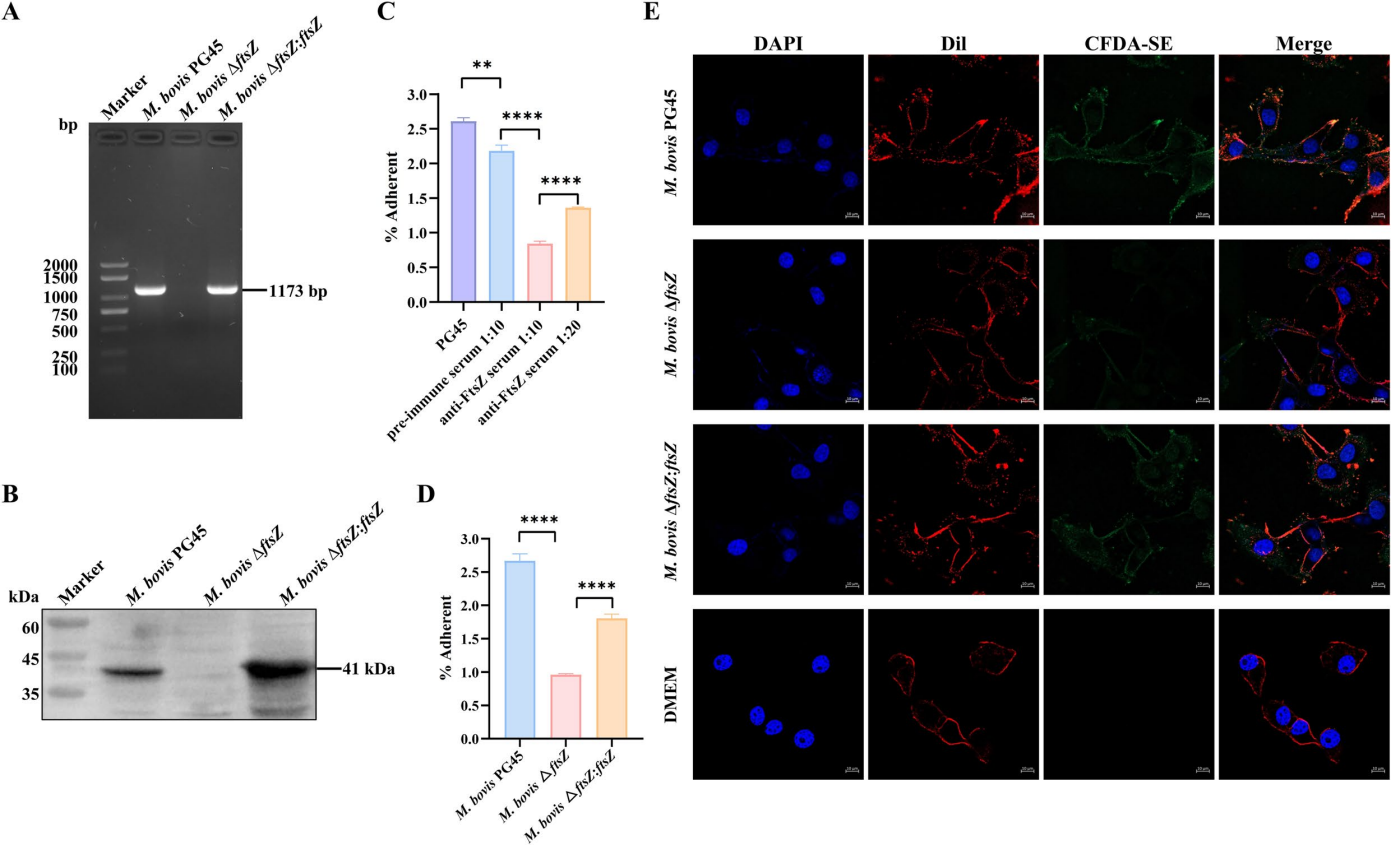
**The destruction of the** ***ftsZ*** **gene reduces the adhesion capacity of** ***M. bovis***. **A** The strains were identified by PCR. The whole DNA of PG45, mutant strains, and complement strains were extracted as templates, and PCR were tested using FtsZ specific primers. **B** The expression of FtsZ protein in *M. bovis* PG45, *M. bovis* Δ*ftsZ*, and *M. bovis* Δ*ftsZ*:*ftsZ*. The presence of the FtsZ protein were examined using anti-FtsZ serum.** C** Inhibition of adhesion to anti-FtsZ serum to *M. bovis.* Different dilutions of heat-inactivated anti-FtsZ serum was preincubated with *M. bovis* PG45 cells at 37 °C for 1 h, while preimmune serum was utilized as a negative control. The adhesion rates were counted and analyzed. **D** The adhesion rates of the strains to EBL cells. *M. bovis* PG45, *M. bovis* Δ*ftsZ*, and *M. bovis* Δ*ftsZ*:*ftsZ* were used to infect EBL cells for 1.5 h and the adhesion rates were counted and analyzed. **E** The adhesion ability of *M. bovis* PG45, *M. bovis* Δ*ftsZ*, and *M. bovis* Δ*ftsZ*:*ftsZ* to EBL cells were observed by laser scanning confocal microscopy. *M. bovis* strains were infected with EBL cells, followed by staining with CFDA-SE, with the nucleus labeled using DAPI and the cell membrane marked with DiI. Red represents the cell membrane, green denotes *M. bovis*, and blue represents the nucleus (scale bar = 10 µm).

The Δ*ftsZ* mutant strain and the complement strain *M. bovis* Δ*ftsZ*:*ftsZ* was confirmed by PCR (Figure 6A). The expression levels of *ftsZ* gene in the Δ*ftsZ* mutant strain and the complement strain *M. bovis* Δ*ftsZ*:*ftsZ* was verified by western blot analysis using anti-FtsZ serum (Figure 6B). The ability of *M. bovis* PG45, *M. bovis* Δ*ftsZ*, and *M. bovis* Δ*ftsZ*:*ftsZ* strains adhesion to EBL cells were subsequently examined by plate counting and visualized by laser confocal microscopy. The Δ*ftsZ* mutant was significantly less adherent to EBL cells than wild-type strain PG45, and this adhesive property was partially restored in the *M. bovis* Δ*ftsZ*:*ftsZ* complement strain (Figure 6D), a similar result was observed under a laser confocal microscope assay (Figure 6E). To further evaluate the specificity of adhesion, we conducted an adhesion inhibition experiment using anti-FtsZ serum. After blocking with anti-FtsZ serum, the adherence ability of *M. bovis* strain to EBL cells decreased significantly compared with the untreated and preimmune serum treated group, and this inhibition of adhesion was dose-dependent, 1:10 dilution of anti-FtsZ serum showed a greater level of adhesion inhibition than the 1:20 dilution (Figure 6C). These above results highlight the pivotal role of FtsZ in *M. bovis* adhesion to EBL cells in bacterial level.

## Discussion

FtsZ, which is commonly considered as a cell division protein, mainly promotes division by regulating the formation of new cell wall peptidoglycan. Compared with other prokaryotic pathogenic bacteria, the most prominent feature of the *Mycoplasma* are the lack of a cell wall. We successfully constructed the *M. bovis* Δ*ftsZ* mutant strain [20], indicating that FtsZ was dispensable in *M. bovis* and there were other cell division compensation mechanisms in this bacterium. This was similar to previous reports that *M. genitalium* is viable in the absence of the FtsZ protein [9, 29].

Understanding the interaction between *M. bovis* and its host is crucial for developing efficient vaccines and drugs that target the critical infection processes, for *Mycoplasma* lacks a cell wall, and the cell membrane is the direct part for its direct interaction with the host cells. Most membrane-associated proteins in *Mycoplasma* can stimulate various lymphocytes to secrete multiple proinflammatory factors, which in turn act on white blood cells to cause tissue damage, thus having good immunogenicity [30]. Therefore, membrane-associated proteins are regarded as potential vaccines and drug targets for *Mycoplasma*. In our research, it was found that the FtsZ of *M. bovis* was mainly distributed in the cell membrane and could react with the serum of infected and immunized cattle, indicating that *M. bovis* infection could stimulate cattle to produce specific antibodies against FtsZ. The amino acid sequence of FtsZ was highly conserved in *M. bovis* strains. These results indicates that FtsZ has the potential to serve as a vaccine target for *M. bovis*.

In addition to good immunogenicity, membrane-associated proteins can also promote adhesion and colonization to the host, which lay a foundation for further invasion and survival. In the previous studies, gene disruption of lipoprotein LppA [12] or MbfN [14] could reduce the adherence capability of *M. bovis* to host cells. Some multifunctional proteins, such as fructose-1,6-bisphosphate aldolase [17] and *α*-Enolase [18], were also found to be distributed on the cell membrane of *M. bovis* and play important role in promoting adhesion. These proteins were identified as adhesion-related proteins of *M. bovis* and some other proteins may be functional in the *M. bovis* adhesion. In this study, rFtsZ could adhere to host EBL cells under laser confocal microscopy and the dose-dependent binding ability between rFtsZ and EBL cell membrane was found through the micro-plate assay. To confirm the adhesion property at the bacterial level, the adhesion of *M. bovis* PG45, *M. bovis* Δ*ftsZ*, and *M. bovis* Δ*ftsZ:ftsZ* to EBL cells were compared. The adhesion capacity of *M. bovis* Δ*ftsZ* to EBL cells was significantly reduced and this adhesive property was partially restored in the *M. bovis* Δ*ftsZ*:*ftsZ* complement strain. Moreover, anti-FtsZ serum could significantly reduce *M. bovis* adhesion to EBL cells. These results indicate that FtsZ act as a novel adhesive-related protein in *M. bovis.*

During the adhesion of pathogenic bacteria, the host extracellular matrix components (ECM) form a physical barrier to defend against infection. However, pathogenic bacteria have evolved various components that hijack ECM to promote adhesion, invasion, and spread, which turns ECM into a pathogen adhesion receptor, thereby promoting infection [31, 32]. At present, components that degrade ECM have been found in various bacteria, such as CollageN Adhesin (CNA) from *Staphylococcus aureus*, which interacts with the triple helix of collagen and promotes host invasion [33]. The histidine triad proteins HtpsC in *Streptococcus suis* has been identified as an adhesive protein, binding fibronectin and laminin through the C-terminal leucine-rich repeat (LRR) domain and the N-terminal histidine trimer domain (HTP) [34]. In previous studies, proteins binding to ECM components were also found in *M. bovis*, indicating that they could also promote infection by interaction with ECM components [14, 35]. In this study, we found that rFtsZ could bind fibronectin, collagen IV, laminin, and vitronectin in a dose-dependent manner. It indicated that the key role of FtsZ in the adhesion of *M. bovis* to host cells through its interaction with ECM components. At the same time, it provides ideas for anti-infection strategies targeting ECM-pathogen interactions.

Plasminogen (plg) is the precursor enzyme of plasmin and is a component in the fibrinolytic system. Plg is widely expressed in extrahepatic tissues such as the spleen, kidneys, and brain [36]. The main function of the plg system is to degrade fibrin clots, connective tissues, and ECM proteins in copy with tissue injury. However, the hijacking of the Plg system by pathogenic microorganisms is believed to promote cross-host epidermal/endothelial migration and spread in tissues owing to the accelerated degradation of ECM [37]. Although plg is not the main host receptor that directly interacts with pathogenic bacterial proteins, plg is considered as a auxiliary factor promoting pathogenic bacteria to binding or infection. Many pathogens express proteins that can bind and enhance plasminogen activity [38]. Pathogens can use the host’s fibrinolytic system to help them invade and colonize [39, 40]. For example, glyceraldehyde-3-phosphate dehydrogenase (GAPDH) and enolase in *Mycoplasma hyorhinis*, as plasminogen receptors (PlgRs), bind and activate plasminogen, assisting the pathogen in degrading the host’s ECM [41, 42]. This study revealed the role of *M. bovis* FtsZ in hijacking the plasminogen system and determines the interaction between FtsZ and plasminogen. rFtsZ could bind plasminogen and plasminogen activator tPA, and promote tPA to activate plasminogen into plasmin, which then degrades plasminase-specific substrates. The lysine analog ε-ACA impeded the activation of plasminogen by FtsZ, indicating that lysine residues were an important functional site for FtsZ to promote the conversion of plasminogen to plasminase. This study indicates that FtsZ is not only a crucial adhesion protein in *M. bovis*, but also can promote pathogen dissemination.

In conclusion, this study revealed FtsZ as a novel adhesion protein with good immunogenicity in *M. bovis*. FtsZ could bind various host ECM and plasminogen, promoting the adhesion and spread of *M. bovis* during infection. These findings not only expand our understanding of FtsZ in the adhesion of *M. bovis*, but also provide a new prospective vaccine and drug candidate for this economically important disease.

## Supplementary Information

**Additional file 1. Plasmids, oligonucleotides, cell lines and strains used in this study.**

**Additional file 2. List of information of different** ***M. bovis*** **strains. Geographical location, collection date, host, isolation source and GenBank of different** ***M. bovis***.

**Additional file 3. Western blot analysis of rFtsZ**. A Western blot analysis of rFtsZ was performed with His-tag mouse monoclonal antibody (1:20,000). B Western blot analysis of rFtsZ were performed with pre-immune mouse serum and anti-FtsZ mouse serum (1:1000).

## Data Availability

The data supporting the findings of this study are available within the articles and the supplementary material.
