# Supplementary figures and images for: FtsZ contributes to cytoadhesion and interaction with host extracellular matrix components and plasminogen in Mycoplasma bovis
Source: Vet Res. 2025 Dec 12;56:228. doi: 10.1186/s13567-025-01673-y (PMC12706882; doi:10.1186/s13567-025-01673-y)

**A**

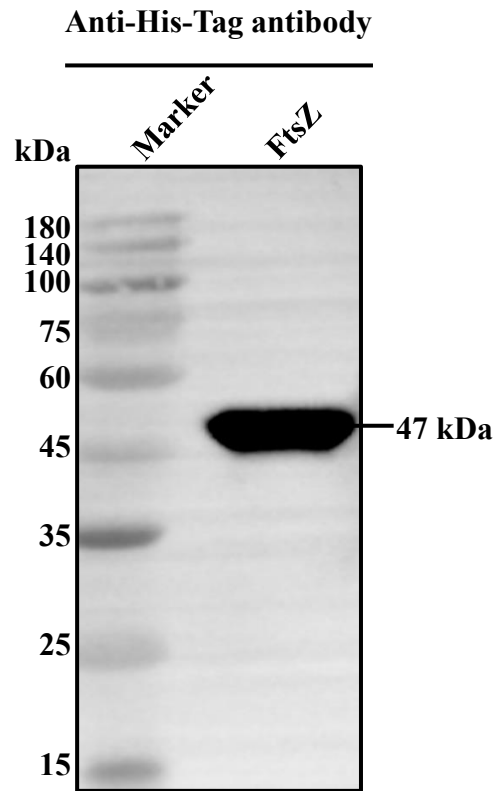

**B**

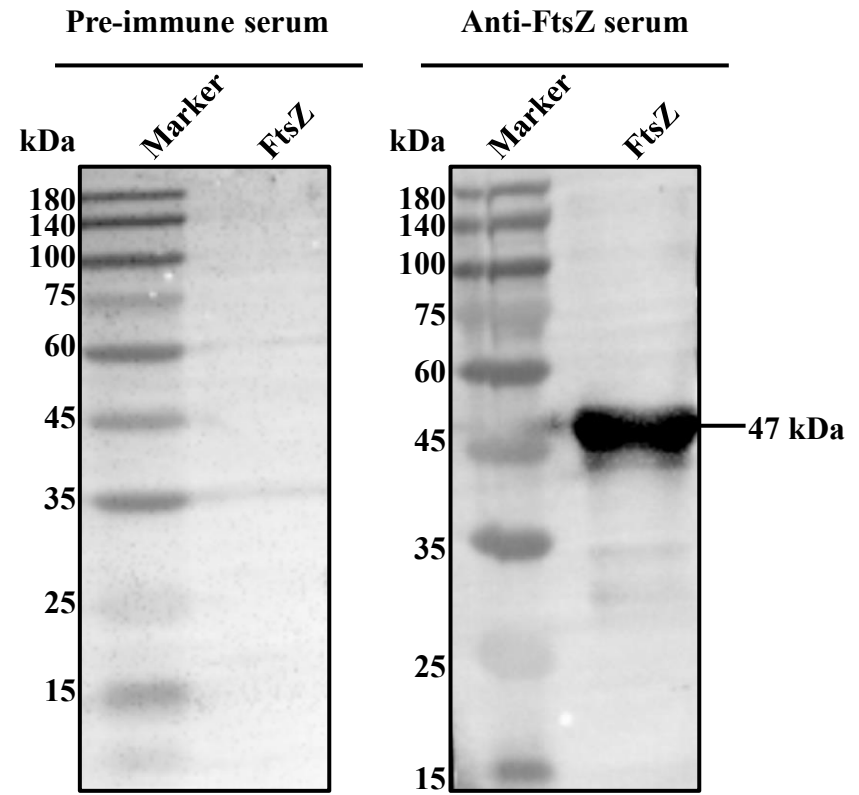

Supplement: Supplementary file 3 — Additional file 3. Western blot analysis of rFtsZ. A Western blot analysis of rFtsZ was performed with His-tag mouse monoclonal antibody (1:20,000). B Western blot analysis of rFtsZ were performed with pre-immune mouse serum and anti-FtsZ mouse serum (1:1000). [file 13567_2025_1673_MOESM3_ESM.pdf]
